# A Boy with a Novel Variant in *TCF20*: An Expanded Phenotype and a Brief Review of the Literature

**DOI:** 10.3390/children12111543

**Published:** 2025-11-14

**Authors:** Diletta Ziveri, Carlo Alberto Cesaroni, Gianluca Contrò, Stefano Giuseppe Caraffi, Francesca Ormitti, Lucrezia Giannini, Agnese Pantani, Anna Cavalli, Susanna Rizzi, Marzia Pollazzon, Daniele Frattini, Carlo Fusco

**Affiliations:** 1 Child Neurology and Psychiatry Unit, Dipartimento Materno-Infantile, Presidio Ospedaliero Santa Maria Nuova, AUSL-IRCCS di Reggio Emilia, 42123 Reggio Emilia, Italy; diletta.ziveri@gmail.com (D.Z.); agnese.pantani@ausl.re.it (A.P.); anna.cavalli@ausl.re.it (A.C.); daniele.frattini@ausl.re.it (D.F.); carlo.fusco@ausl.re.it (C.F.); 2 Laboratorio di Neurofisiologia Pediatrica, Dipartimento Materno-Infantile, Azienda USL-IRCCS Di Reggio Emilia, 42123 Reggio Emilia, Italy; 3 Medical Genetics Unit, Azienda USL-IRCCS di Reggio Emilia, 42123 Reggio Emilia, Italy; gianluca.contro@ausl.re.it (G.C.); stefanogiuseppe.caraffi@ausl.re.it (S.G.C.); lucrezia.giannini@ausl.re.it (L.G.); marzia.pollazzon@ausl.re.it (M.P.); 4 Neuroradiology Unit, University Hospital of Parma, 43126 Parma, Italy; 5 Medical Genetics Unit, IRCCS Azienda Ospedaliera Universitaria di Bologna, 40138 Bologna, Italy

**Keywords:** *TCF20*, ADHD, Chiari I malformation, oculomotor dyspraxia

## Abstract

**Highlights:**

**What are the main findings?**

- Chiari I Malformation.
- Oculomotor dyspraxia.

**What are the implications of the main findings?**

- Expanding neuroradiological findings in *TCF20*-NDD.
- Exome sequencing is extremely important when the clinical picture is unclear.

**Abstract:**

**Background:** *TCF20*-associated neurodevelopmental disorder (*TCF20*-NDD) is a heterogeneous clinical condition resulting from defects in gene-encoding Transcription Factor 20, which plays a key role in neuronal development and synaptic function. Here, we present a novel case involving an 11-year-old boy who was referred to us for a neuro-developmental disorder characterized by attention deficit hyperactivity disorder (ADHD), tremor in the upper limbs, tilted head posture, motor delay, impaired executive functioning, and oculomotor dyspraxia. **Methods:** Genetic tests were performed, including CGH array, molecular analysis of the FMR1 gene, molecular analysis using a next-generation sequencing gene panel targeted for spinocerebellar diseases, and finally, WES including mitochondrial genome analysis. A neuroimaging study of brain and spine was performed using MRI. **Results:** Trio Whole Exome Sequencing revealed a de novo pathogenic frameshift variant NM_001378418.1:c.5009dup, p.(Thr1671Aspfs*5) in the *TCF20* gene. The MRI scan of the brain, cervical, dorsal, and lumbosacral spine revealed Chiari type I malformation. Regarding the pathogenic mechanism underlying Chiari I malformation, it could be found in the homology between TCF20 and the RAI1 gene, the latter being associated with alterations in the posterior cranial fossa. **Conclusions:** We emphasize the use of exome sequencing in patients with unclear clinical presentations, with awareness of *TCF20*-associated neurodevelopmental disorder; paying attention to brain MRI findings would be useful to further expand the phenotype of *TCF20*-NDD.

## 1. Introduction

The *TCF20* gene (OMIM *603107), located on chromosome 22q13.2, encodes Transcription Factor 20 (TCF20), previously known as stromelysin-1 platelet-derived growth factor-responsive element binding protein (SPBP) or AR1 [1,2]. TCF20 shows widespread expression in brain tissue, especially in the hippocampus and cerebellum [3,4,5] and has been recently found to be part of a chromatin regulatory complex that also includes MeCp2, PHF14, RAI1, and HMG20A. These proteins share the function of regulating chromatin and gene expression, specifically for neuronal development and synaptic function [6].

Pathogenic variants in the *TCF20* gene have been identified in over 100 patients and are associated with autosomal dominant developmental delay with cognitive impairment and behavioral abnormalities (TCF20-NDD). Causative variants are usually de novo, though a few have been inherited from an affected parent [7]. A total of 131 *TCF20* variants are annotated as pathogenic or likely pathogenic in the ClinVar database (accessed 5 November 2025) [8], 128 of which are single nucleotide variants or small delins resulting in a premature stop codon and a loss-of-function (LoF). The LOEUF score (loss-of-function observed/expected upper bound fraction) value of the *TCF20* gene is 0.05, indicating that the gene is intolerant to LoF variants and haploinsufficiency is the main disease mechanism [5,9]; a few missense variants were also described, but no functional studies were performed to ascertain their effect [9]. Some cases also show structural variants such as deletions or inversions; duplications with a minimal common region centered on *TCF20* have also been associated with NDD, suggesting that the precise dosage of this transcription factor may be crucial for proper brain development [6,7].

The phenotype of *TCF20*-NDD is broadly heterogeneous and may include intellectual disability, autism spectrum disorder, hyperactivity, hypotonia, craniofacial dysmorphisms, seizures, somatic overgrowth with macrocephaly, sleep disturbances, and movement disorders [1,5,6,7].

Autism spectrum disorder is one of the most common features [10,11,12].

A few individuals presented with movements disorders, such as tremors and dyskinesia/jerky movements [13,14,15].

Here, we report a boy with Chiari malformation type I, severe dyspraxia, and motor delay, associated with a novel, de novo, frameshift variant in *TCF20.*

## 2. Case Report

The patient is an 11-year-old boy, who is the firstborn son of apparently healthy and unrelated parents.

The family history is negative for neurodevelopmental disorders or Chiari malformation type I. The patient was born at 40 weeks by cesarean section for breech presentation. During pregnancy, fetal movements were defined as hypovalid.

At birth, Apgar scores were four and nine at 1 and 5 min, respectively. Perinatal adaptation was good except for the presence of jaundice, which lasted several months and was treated with phototherapy.

He achieved independent sitting at 15 months, independent walking at 18 months, and babbling at 10 months; first words were at around one year of age, with speech articulation disorder. Motor clumsiness has been present since the first months of life, as well as sialorrhea. His gross motor skills have improved over time, though at the last examination he still had difficulties with fine motor skills. A transfontanellar ultrasound was performed after birth and showed the presence of a germinative cyst and choroid plexus cysts. Arts syndrome was excluded via molecular analysis of the *PRPS1* gene.

The patient underwent day hospital admission at the age of one year due to a tilted and rotated head posture. MRI of the brain and MRI of the cervical, dorsal, and lumbosacral spine performed during admission revealed ectopia of the cerebellar tonsils.

**Figure 1:**
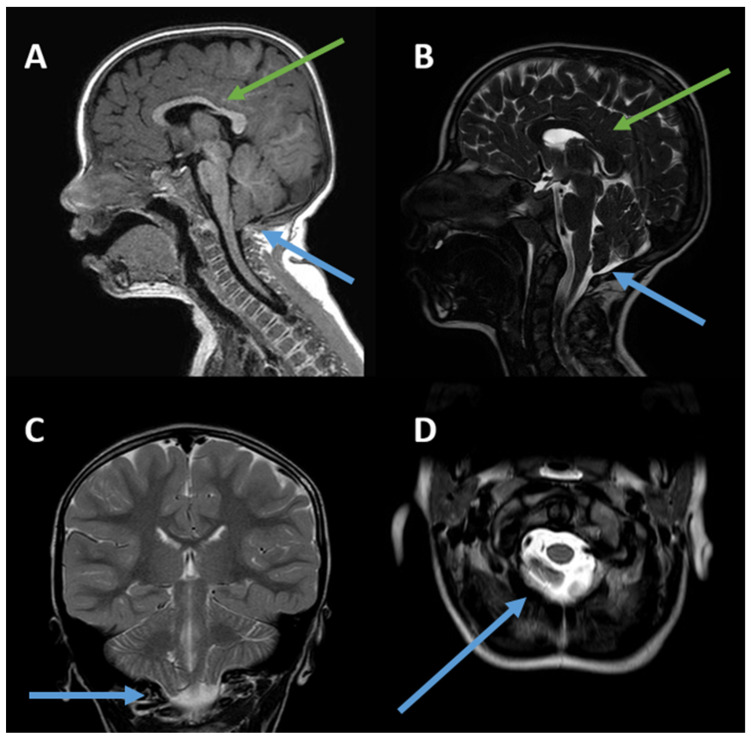
(**A**–**D**). Chiari I malformation and slightly thinned appearance of the body/splenion junction of the corpus callosum. (**A**) Sagittal 3D TFE T1-weighted image; (**B**) sagittal T2-weighted image; (**C**) coronal T2-weighted image; (**D**) axial T2-weighted image. The green arrows indicate thinning of the corpus callosum, while the blue arrows refer to Chiari I malformation. The posterior fossa is small. The peg-like cerebellar tonsils (**C**) herniate into the foramen magnum (**D**). Tonsillar ectopia is more pronounced on the right (**C**). The axial image shows the tonsils (**D**) behind the medulla oblongata, which cause crowding of the foramen magnum.

He was hospitalized again at the age of 6 years for motor impairment, sialorrhea, oral dyspraxia, and upper-limb tremors, which were reported since the age of 4 years. At this time, a second brain MRI confirmed ectopia of the cerebellar tonsils in the retro-medial cerebrospinal fluid space and through the foramen magnum, consistent with Chiari type I malformation, and reported a slight deviation of the optic chiasm, downward and to the right (Figure 1). The patient was discharged with a diagnosis of ataxia and a speech articulation disorder, as well as ectopia of the cerebellar tonsils (6 mm).

During follow-up, an EEG showed posterior slow activity of non-specific significance.

A nerve conduction study examination showed normal results.

Neuropsychological evaluation performed at the age of 10 years revealed impaired fluid intelligence associated with visuospatial difficulties. Data collected during the assessment indicated visuospatial deficits in a subject with mixed laterality, short-term memory within normal limits, good working memory, impaired language comprehension with adequate expressive abilities, unstable attention, and executive impulsivity. In the learning domain, writing skills were appropriate; however, reading decoding was slow, with difficulties in comprehension of the presented tasks and in mathematical activities.

In light of his unstable attention span and impulsiveness, diagnostic tests were performed for attention deficit hyperactivity disorder (ADHD). The diagnosis of ADHD was made at the local Child Neuropsychiatry Center with territorial jurisdiction. Based on medical history and clinical interviews, questionnaires, observation scales, and neuropsychological tests assessed attention and executive functions.

The most recent neurological examination at the age of 11 years revealed a clumsy gait, without signs of ataxia, and a tendency to walk on tiptoe. There was also evidence of severe ocular dyspraxia and difficulty following a target. Deep tendon reflexes were brisk and symmetrical. Physical examination revealed a regular head shape, slightly up-slanting palpebral fissures, wide mouth, and regular pectus. There was asymmetry of the scapulae, with the right lower than the left.

Molecular analysis of the FMR1 gene yielded results in the normal range. The CGH array test revealed a partial duplication of the long arm of chromosome 17 inherited from the mother, which was not causative for the condition described and was considered a variant of uncertain significance.

Molecular analysis using a next-generation sequencing gene panel targeted for spinocerebellar diseases revealed a heterozygous variant of uncertain significance in the *CCDC88C* gene, NM_001080414.4:c.2090G>C, p.(Arg697Pro). While biallelic LoF variants in this gene are known cause of congenital hydrocephalus (OMIM #236600), two different missense variants have been described in a large family with autosomal dominant spinocerebellar ataxia [16] and in one individual with early onset pure hereditary spastic paraplegia [17], but a formal association with these phenotypes is still pending. Moreover, the variant identified in our patient is considered to be tolerated by in silico predictions (REVEL score 0.054) and reported as heterozygous in 13 individuals (12 older than 50 years) in the population database gnomAD v4.1.0 (accessed 5 November 2025).

Further genetic investigations via trio Whole Exome Sequencing, including mitochondrial genome analysis, identified the de novo variant NM_001378418.1:c.5009dup, p.(Thr1671Aspfs*5) in the *TCF20* gene, classified as pathogenic according to ACMG recommendations [18] (null variant, de novo with parenthood confirmed, absent in population database gnomAD v4.1.0 accessed 5 November 2025). WES was negative for analysis of del/dup (CNV).

## 3. Discussion

We describe a case with neurodevelopmental disorder, severe dyspraxia, and a novel pathogenic variant in the *TCF20* gene. The most notable aspect of this patient’s condition is the presence of severe dyspraxia in both manual and motor activities, as well as in the oculo-motor domain. In particular, there are descriptions of abnormalities in saccadic movements and nystagmus [9,19]. At first, the hypothesis formulated for these patients at the onset of symptoms was spinocerebellar ataxia. Upon arrival at our center, a genetic panel for ataxia had already been performed, with negative results. In fact, the tremor in the upper limbs, initially reported as a cerebellar tremor, improved over time, although severe dyspraxia persisted. The molecular diagnosis emerged instead from Whole Exome Sequencing.

Individuals with dyspraxia have already been described in the literature. For example, in the article by Vetrini et al. [9], six patients with motor coordination disorders were described, two of whom also had ocular involvement, as in our patient. There are also patients with TCF20 who exhibit abnormal movements that can mimic much more serious conditions; while in our case the initial differential diagnosis was spinocerebellar ataxia, Prasun and colleagues were faced with a patient with dystonic cerebral palsy-like presentation [14].

Neuroimaging examinations in our patient revealed a Chiari type I malformation with descent of the cerebellar tonsils in all proposed projections. The descent of the cerebellar tonsils by 6 mm meets the minimum criterion for the diagnosis of type 1 Chari syndrome [20,21]. There are no other reported cases of Chiari I malformations in *TCF20*-NDD. However, the *TCF20* gene is homologous to the *RAI1* gene [5,9] which is associated with Smith–Magenis syndrome (SMS), a condition that may include posterior cranial fossa alterations [22]. Specifically, one SMS patient was described as having a small brain stem and vermis, as well as a mega cisterna magna in the posterior fossa. Neuroimaging examinations in our patient revealed thinning of the corpus callosum, in disagreement with a case series proposed by Schneeweiss et al. in 2022 [23], in which two adolescents with a thickened corpus callosum were described. In fact, the *TCF20* gene may be indirectly involved in neuronal migration processes, as well as the development of the corpus callosum. In 2022, Zhou et al. [7] described a strong interaction between TCF20 and MeCP2 in the development of brain structures, highlighting its fundamental interactive role in preventing morphological alterations. Indeed, the *MECP2* gene plays a role in the development of the corpus callosum, influencing its hypoplasia. This has been demonstrated in both animal and human models using voxel-based morphometry and tract-based spatial statistics [24,25].

For a neuroradiological picture comparable to those described in the literature for patients with TCF20 mutation, refer to Table 1 for comparison with other cases of TCF20-related syndrome in which alterations were detected on brain MRI.

In Chiari type I malformation, as described in the literature, ataxia-like symptoms are typically due to direct compression of the cerebellar vermis and hemispheres [28,29,30,31]. In light of this, it is unlikely that the upper-limb tremors, motor impairment, and walking difficulties reported in our patient are due to a direct effect of Chiari malformation. In particular, there were no evident signs of compression of the brainstem or upper spinal cord (Figure 1). To clarify the picture described above, we have added a table to the Supplementary Materials (Table S1) showing the symptoms associated with Chiari malformation.

Indeed, according to the DSM-5-TR [32] diagnosis, in order to diagnose dyspraxia, it must have an impact on quality of life, there must be significant motor difficulties (with difficulty acquiring new skills), and there must be no conditions that could predispose the individual to developing dyspraxia. Considering the diagnosis of neurodevelopmental disorders related to TCF20, it would be more appropriate to refer to it as a co-occurring developmental coordination disorder (co-occurring DCD). We attached the criteria for co-occurring DCD in Supplementary Table S2.

## 4. Take-Home Messages

- Further studies are essential to delineate potential associations between TCF20 gene variants and MRI-detectable alterations. At present, based on the available data, it is not possible to identify neuroradiological features that are specific to this condition.
- Motor impairment is a feature commonly associated with TCF20 gene alterations; in our case, however, the Chiari I malformation does not appear to be severe enough to contribute significantly to the patient’s symptoms.
- When the etiology of a child’s motor impairment is unclear, particularly in the presence of ataxia-like features such as upper-limb tremors, it is advisable to perform comprehensive genetic testing to avoid overlooking potential diagnoses.

## Figures and Tables

**Table 1:** Pathologic neuroimaging findings in individuals with TCF20 NDD.

Reference/(Case #)	*TCF20* Variant (NM_001378418.1)	Pathologic Neuroimaging Findings
[23] (B)	c.1261A>T, p.(Thr421Ser)	Corpus callosum had an abnormal appearance with overall thickening and slightly lobulated contour, likely reflecting underlying abnormalities in the organization of the white matter.
[5]	c.1839_1872del, p.(Met613Ilefs*159)	Symmetric patchy abnormal signals in the bilateral parietal white matter and adjacent to the bilateral lateral ventricles, and insufficient cerebral white matter myelination were observed. The nuclei in the bilateral basal ganglia showed symmetric, slightly short T1 and slightly short T2 signals; the bilateral lateral paraventricular region and bilateral parietal white matter revealed symmetric patchy, slightly long T1 and slightly long T2 signals, isointense signals on fluid attenuated inversion recovery (FLAIR) image, and slightly low-intense signals with indistinct edges on diffusion weighted imaging (DWI).
[9] (#6)	c.2327_2328del, p.(Gln776Argfs*5)	Mild cerebellar atrophy.
[9] (#16)	c.4894del, p.(Tyr1632Thrfs*6)	Not specified.
[9] (#23)	c.5652_56553del, p.(Glu1884Aspfs*31)	Not specified.
[9] (#26)	c.5719C>T, p.(Arg1907*)	Delayed CNS myelination and lack of cerebral white matter.
[10,11,26,27]	NA	Abnormality of the cerebrum *n* = 2/12 (not better specified).

## Data Availability

The data presented in this study are available on request from the corresponding author for privacy reasons.

## Supplementary Materials

The following supporting information can be downloaded at: https://www.mdpi.com/article/10.3390/children12111543/s1, Table S1: Symptoms associated with Chiari I malformation. Table S2. Criteria for co-occurring DCD. Refs. [33,34,35].
