# Supplementary material for: A Boy with a Novel Variant in TCF20: An Expanded Phenotype and a Brief Review of the Literature
Source: Children (Basel). 2025 Nov 14;12(11):1543. doi: 10.3390/children12111543 (PMC12651428; doi:10.3390/children12111543)
Supplement: Supplementary file 1 [file children-12-01543-s001.zip › children-3947239-supplementary.pdf]

Table S1. Symptoms associated with Chiari Malformation Type I.

Category	Typical Symptoms	Comments / Notes
**Headache and neck pain**	Occipital / suboccipital headache, often triggered or worsened by coughing, sneezing, or straining (Valsalva maneuver)	Considered one of the most frequent symptoms.
**Balance / motor disturbances**	Dizziness, unsteadiness, ataxia, difficulty walking	Compression of the cerebellum, brainstem, or upper spinal cord can generate these symptoms
**Sensory disturbances**	Paresthesias (tingling, numbness), loss of temperature or pain sensation, "cape-anesthesia" (mantle-like area) in case of associated syringomyelia	The spinal cord may be involved, particularly when syringomyelia is present.
**Visual / auditory disturbances**	Double vision (diplopia), nystagmus, light sensitivity, tinnitus, hearing loss (hypoacusis)	These may result from brainstem involvement or compression of cranial nerves.
**Dysphagia / dysarthria / cranial nerve problems**	Difficulty swallowing, hoarse voice, weakness of lower cranial nerves (IX, X, XI, XII)	Less frequent, but reported in cases with compression of the medulla or lower cranial nerves.
**Other neurological / autonomic symptoms**	Sleep apnea, syncope, thermoregulation disturbances, scoliosis (when associated with syringomyelia)	Variability is high: some cases are asymptomatic, while others present more complex manifestations.

Reference Table S1.

- Nash J, Cheng JS, Meyer GA, Remler BF. Chiari type I malformation: overview of diagnosis and treatment. WMJ. 2002;101(8):35-40.
- McClugage SG, Oakes WJ. The Chiari I malformation. J Neurosurg Pediatr. 2019 Sep 1;24(3):217-226. doi: 10.3171/2019.5.PEDS18382. Epub 2019 Sep 1.
- Tubbs RS, Lyerly MJ, Loukas M, Shoja MM, Oakes WJ. The pediatric Chiari I malformation: a review. Childs Nerv Syst. 2007 Nov;23(11):1239-50. doi: 10.1007/s00381-007-0428-0.
- Kular S, Cascella M. Chiari Malformation Type 1. 2024 Feb 9. In: StatPearls [Internet]. Treasure Island (FL): StatPearls Publishing; 2025 Jan–.

Table S2. Criteria for co-occurring DCD.

Criterion	Description	Specific Notes for Co-occurring DCD
**A**	The coordination motor skills are substantially below those expected for the chronological age, interfering with daily or academic activities.	The child shows marked motor deficits compared to peers, with clear functional impact.
**B**	The motor difficulties significantly interfere with daily or academic activities, causing functional limitations.	Even in the presence of another medical/genetic condition, the motor problems limit active participation.
**C**	The motor difficulties are not better explained by intellectual disability, visual impairment, or neurological conditions such as cerebral palsy, muscular dystrophy, or degenerative disease.	For co-occurring DCD, motor difficulties are not fully attributable to the concurrent genetic or neurological condition.
**D**	If a neurological or genetic condition is present, the motor difficulties are more severe than would be expected for that condition.	Diagnosis of co-occurring DCD is made only if the motor deficit is disproportionate to the primary clinical condition.

Reference Table S2.

- American Psychiatric Association. (2022). Diagnostic and statistical manual of mental disorders (5th ed., text rev.). Washington, DC: American Psychiatric Association Publishing.
